# Gene Amplification as a Mechanism of Yeast Adaptation to Nonsense Mutations in Release Factor Genes

**DOI:** 10.3390/genes12122019

**Published:** 2021-12-19

**Authors:** Evgeniia M. Maksiutenko, Yury A. Barbitoff, Andrew G. Matveenko, Svetlana E. Moskalenko, Galina A. Zhouravleva

**Affiliations:** 1 Department of Genetics and Biotechnology, St. Petersburg State University, 199034 St. Petersburg, Russia; st035219@student.spbu.ru (E.M.M.); st035189@student.spbu.ru (Y.A.B.); a.matveenko@spbu.ru (A.G.M.); s.moskalenko@spbu.ru (S.E.M.); 2 St. Petersburg Branch, Vavilov Institute of General Genetics of the Russian Academy of Sciences, 199034 St. Petersburg, Russia; 3 Bioinformatics Institute, 197342 St. Petersburg, Russia; 4 Laboratory of Amyloid Biology, St. Petersburg State University, 199034 St. Petersburg, Russia

**Keywords:** translation termination, release factors, yeast, eRF1, eRF3, nonsense suppression, amplification, plasmid copy number, chromosome instability, WGS

## Abstract

Protein synthesis (translation) is one of the fundamental processes occurring in the cells of living organisms. Translation can be divided into three key steps: initiation, elongation, and termination. In the yeast *Saccharomyces cerevisiae*, there are two translation termination factors, eRF1 and eRF3. These factors are encoded by the *SUP45* and *SUP35* genes, which are essential; deletion of any of them leads to the death of yeast cells. However, viable strains with nonsense mutations in both the *SUP35* and *SUP45* genes were previously obtained in several groups. The survival of such mutants clearly involves feedback control of premature stop codon readthrough; however, the exact molecular basis of such feedback control remain unclear. To investigate the genetic factors supporting the viability of these *SUP35* and *SUP45* nonsense mutants, we performed whole-genome sequencing of strains carrying mutant *sup35-n* and *sup45-n* alleles; while no common SNPs or indels were found in these genomes, we discovered a systematic increase in the copy number of the plasmids carrying mutant *sup35-n* and *sup45-n* alleles. We used the qPCR method which confirmed the differences in the relative number of *SUP35* and *SUP45* gene copies between strains carrying wild-type or mutant alleles of *SUP35* and *SUP45* genes. Moreover, we compare the number of copies of the *SUP35* and *SUP45* genes in strains carrying different nonsense mutant variants of these genes as a single chromosomal copy. qPCR results indicate that the number of mutant gene copies is increased compared to the wild-type control. In case of several *sup45-n* alleles, this was due to a disomy of the entire chromosome II, while for the *sup35-218* mutation we observed a local duplication of a segment of chromosome IV containing the *SUP35* gene. Taken together, our results indicate that gene amplification is a common mechanism of adaptation to nonsense mutations in release factor genes in yeast.

## 1. Introduction

Nonsense mutations are point mutations which lead to the appearance of premature termination codons (PTC) that cause untimely translation termination and consequently prevent normal full-length protein synthesis. According to various estimates from 11 to 30% of all life-threatening genetic diseases such as cystic fibrosis, Duchenne muscular dystrophy, and cardiac conduction disease are caused by nonsense mutations [1,2,3,4,5]. At the same time, there is a natural mechanism neutralizing the consequences of nonsense mutations by allowing stop codon readthrough. It is called nonsense suppression and takes place in all living cells, from bacteria to mammals [6]. The efficiency of nonsense suppression depends on the functioning of various cellular components. Components of the translational apparatus, tRNAs, ribosomes, protein elongation and termination factors, make the biggest contribution to the nonsense suppression.

Baker’s yeast *Saccharomyces cerevisiae* is commonly used as a model organism for studying translation. Yeast, like all eukaryotes, has two translation termination factors: eRF1 and eRF3. The eRF1 protein belongs to class 1 translation termination factors and is encoded by the essential *SUP45* gene. eRF1 is responsible for the recognition of all three stop codons and performs peptidyl-tRNA hydrolysis [7]. Another release factor, eRF3, is a member of the class 2 termination factor family. Its main function is to stimulate the activity of class 1 factors due to its GTPase activity. In yeast, eRF3 is encoded by the essential *SUP35* gene [8,9,10]. Despite the fact that both *SUP45* and *SUP35* genes are indispensable for yeast cells, viable strains with nonsense mutations in both the *SUP45* genes [11] and *SUP35* [12] were previously obtained in our laboratory and other research groups [13,14,15].

In earlier works, we have characterized a series of spontaneous nonsense mutations in the *SUP45* and *SUP35* genes (*sup45-n* and *sup35-n*) [11,12]. Two of these *sup45-n* mutations (*sup45-101*, *sup45-102*) are located in the region of the *SUP45* gene encoding the NM-domains of eRF1. Three of the mutations (*sup45-104*, *sup45-105*, *sup45-107*) are located in the last third of the *SUP45* gene, encoding the C-terminal domain of eRF1 [11]. All these mutations are situated in a weak termination context. Thus, due to the nonsense suppression all *SUP45* nonsense mutants contain the full-length eRF1 protein, although its level is significantly reduced in strains carrying the *sup45-n* mutant allele as compared to the wild-type strain [11]. Five of the *sup35-n* mutations (*sup35-203*, *sup35-218*, *sup35-244*, *sup35-260*, *sup35-74*) are located in the first third of the *SUP35* gene, encoding the NM-domain of eRF3, and the other mutations (e.g., *sup35-21*) in the region encoding the C-terminal domain of eRF3 [12]. It is necessary to mention that, besides *sup35-n* mutation, the translation termination process is influenced by the aggregation of Sup35 protein. Sup35 is able to form a self-perpetuating amyloid-like aggregates, giving rise to the [*PSI*^+^] prion [16,17]. Aggregation of Sup35 in the [*PSI*^+^] cells results in defective translation termination due to a decreased amount of functional Sup35. Previously, it was shown that the *sup35-n* mutations are synthetically lethal with the [*PSI*^+^] prion, though such lethality depends on the genetic method used for the creation of such a combination [18]. Similarly to *sup45-n*, all *sup35-n* mutants demonstrate strong omnipotent nonsense suppression and produce low levels of full-length eRF3 protein [12].

Multiple effects of mutations in the *SUP35* and *SUP45* genes have been revealed, but their origin and mechanisms leading to viability of nonsense mutations in the essential genes still need to be clarified. One of the hypotheses explaining this phenomenon suggests that cells harbouring *sup45* nonsense alleles are viable due to the establishment of feedback-regulated readthrough of the PTC. Specifically, depletion of the full-length eRF1 promotes tRNA-mediated stop codon readthrough, which, in turn, drives production of the full-length eRF1 [19]. On the other hand, it is also possible that accumulation of additional mutations, which occurs during selection, increases the viability of cells with *sup45* or *sup35* nonsense mutations [11,20]. To investigate the genetic factors supporting the viability of these mutants, we performed whole-genome sequencing of 100 strains carrying mutant alleles *sup35-n* and *sup45-n* using the Illumina technology. Such an analysis revealed an important role of gene amplification as a mechanism of adaptation to translation termination factor mutations in yeast.

## 2. Materials and Methods

### 2.1. Yeast Strains and Media

Standard methods of cultivation and manipulation of yeast were used throughout this work [21,22]. *S. cerevisiae* strains and plasmids used in this work are listed in Table 1 and Table 2, respectively. All the plasmids mentioned in this work are derivatives of centromeric pRS315 (*LEU2*) or pRS316 (*URA3*) vectors [23]. U-1A-D1628 [24] and U-14-D1690 are 1A-D1628 derivatives containing pRS316-SUP45 [11] and pRSU1 [25] plasmids, as a source of the essential *SUP45* or *SUP35* genes, respectively. The U-14-D1690 strain was obtained as follows: first, a Leu^+^ Ura^−^ haploid segregant (14-D1690) was sporulated from diploid obtained via cross between U-1A-D1628 and L-12-D1682 [26]. Then the pRSU1 plasmid was substituted for the pRSU2 [25] by selecting Leu^−^ Ura^+^ strain among 14-D1690 [pRSU2] transformants. Yeast strains were cultivated at 26 °C in standard solid and liquid media: YEPD (rich media) and SC (synthetic media). SC medium containing 1 g/L 5-fluoroorotic acid (Thermo Scientific, Waltham, MA, USA) was used for selection against cells bearing plasmids with the *URA3* marker [22]. For genome sequencing experiments, incubation on complete media was used alongside 5-FOA media to select cells that lost the *URA3* plasmid. Yeast transformation was carried out according to the standard protocol [27].

### 2.2. Genomic DNA Extraction

For genomic DNA extraction, yeast strains were grown in 20 mL of SC medium with additional adenine (final concentration 40 mg/L) until OD_600_ of 1.00. Cells were collected by centrifugation in a 50 mL falcon tube for 10 min at 6000 g, washed with 50 mL of distilled water, and precipitated again. Next, genomic DNA was extracted as described previously [24]. The concentration and quality of genomic DNA (gDNA) were monitored by agarose gel electrophoresis and by optical density measurement using NanoDrop spectrophotometer (Thermo Scientific, Waltham, MA, USA).

### 2.3. Whole-Genome Sequencing

We sequenced genomes of 100 strains harboring wild-type or mutant alleles of the *SUP35* and *SUP45* genes. For each *sup35-n* allele, 8 (in case of *sup35-74* and *sup35-240*) or 9 (in case of *sup35-21* and *sup35-218*) independent colonies were selected via spontaneous plasmid loss. For each *sup45-n* allele, 8 independent colonies were selected using spontaneous plasmid loss and 3 — using 5-FOA containing media. Alongside strains bearing *sup35-n* and *sup45-n* alleles, we sequenced genomes of 4 independent colonies bearing a plasmid containing wild-type *SUP35* allele, and of 7 colonies bearing a plasmid containing wild-type *SUP45* allele (of these, 4 were selected via spontaneous plasmid loss and 3 — using 5-FOA containing media).

Libraries for whole-genome sequencing were prepared as described in [24]. We sequenced the yeast DNA library on Illumina HiSeq 2500 platform in a paired-end mode with the read length of 2 × 150 bp.

### 2.4. Whole-Genome Sequencing Data Analysis

For all subsequent analyses, whole-genome sequencing (WGS) reads were aligned onto the reference genome of the Peterhof genetic collection strain U-1A-D1628 [24] or onto the sequences of pRSU1/pRS315-SUP45 plasmids using the BWA MEM algorithm [31]. Alignments were sorted and indexed using SAMtools [32]. Duplicate reads in each alignment file were marked using the Genome Analysis ToolKit (GATK) v. 4.1 [33]. After marking the duplicate reads, alignment files were used for coverage analysis and variant calling.

For initial coverage analysis, we used qualimap v.2.2.2-dev [34]. For a more detailed analysis, per-base coverage data were collected using SAMtools. Plasmid copy number was estimated by averaging such per-base coverage values across all plasmid-specific sequences using a custom script and then dividing the average value by the mean coverage of a yeast genome.

For small variant analysis, per-sample variant calling was performed using the GATK HaplotypeCaller tool in the ERC GVCF mode. After initial variant calling, joint genotyping of all samples was conducted. For variant calling against the complete genome sequence, variants were annotated using a custom script based on NCBI BLAST+ v.2.9.0 [35] alignment of a genomic region against reference mRNA sets of *S. cerevisiae* downloaded from the Saccharomyces Genome Database (https://yeastgenome.org/, accessed on 19 June 2019). Variant calls were manually curated to exclude calling or assembly artifacts.

### 2.5. RNA Extraction and cDNA Generation

For extraction of RNA, cultures were grown in YEPD liquid medium until OD_600_ of 0.5–0.6 (iMark Absorbance Reader, BioRad, Hercules, CA, USA); then the cells were harvested and washed. Total yeast RNA was isolated using the GeneJET RNA Purification Kit (Thermo Scientific, Waltham, MA, USA, #K0731) and treated with DNase I (RapidOut DNA Removal Kit, Thermo Scientific, Waltham, MA, USA, #K2981) according to the manufacturer’s instructions. RNA concentration and quality were evaluated using a NanoDrop Spectrophotometer (Thermo Scientific, Waltham, MA, USA). Purified RNA was reverse transcribed with RevertAid RT Reverse Transcription Kit (Thermo Scientific, Waltham, MA, USA, #K1691). cDNA generation was performed under the following conditions: 25 °C for 5 min, 42 °C for 60 min and termination at 70 °C for 5 min.

### 2.6. qPCR

The copy number of target genome fragments or the expression level of target genes were analyzed by quantitative PCR (qPCR) with EVA Green 2.5X PCR-mix (Syntol, Moscow, Russia) according to the manufacturer instructions. Primer pairs used in this analysis are listed in Table 3. Reactions and quantification were performed using CFX96 amplifier (Bio-Rad, Hercules, CA, USA). The quantitation cycle for *ACT1* (for genomic DNA) or for *ADH1* (for cDNA) was used as a reference. Triplicate qPCRs were performed for each biological replicate. The ΔΔC_T_ method [36] was used to measure the relative copy number. The resulting values were used to estimate the copy number of plasmid containing *sup35-n* and *sup45-n* or to quantify gene expression changes.

### 2.7. Statistical Analyses and Code Availability

All experiments were done in at least six biological replicates. To evaluate the differences between groups using qPCR data, Wilcoxon rank-sum test was performed using R v.4.1. Differences were considered statistically significant at adjusted *p*-value < 0.05. All code pertinent to the bioinformatic or statistical analysis presented in this work is available at https://github.com/mrbarbitoff/yeast_cnv_analysis/.

## 3. Results

**Figure 1:**
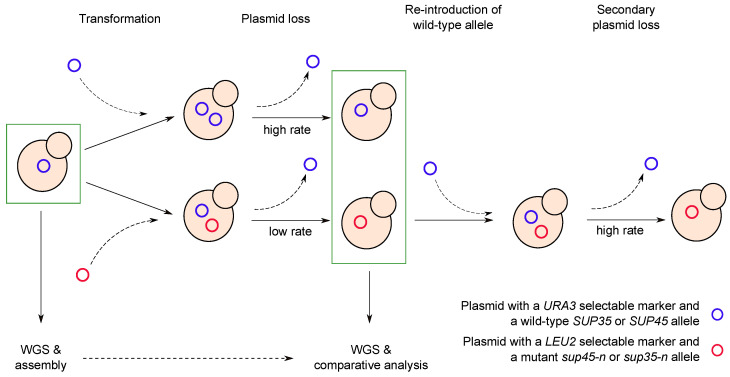
A diagram illustrating the experimental design used to study yeast adaptation to nonsense mutations in release factor genes. Note that yeast cells lose a plasmid containing wild-type release factor allele at much higher rate during secondary loss (after re-introduction of the wild-type allele), indicating accumulation of genetic or epigenetic changes in the strains during the initial plasmid shuffling event.

The very existence of viable nonsense mutations in the essential release factor genes is unexpected; however, it is also important that yeast cells are capable of adapting to these mutations. Such an adaptation can be observed in the following experiment performed by Moskalenko et al. [11,20] (Figure 1): yeast cells with a deletion of a normal chromosomal copy of either *SUP35* or *SUP45* and bearing a wild-type allele of the corresponding gene on a plasmid are transformed with a second plasmid bearing either a wild-type or mutant allele of the same gene. After this initial transformation, yeast cells are grown on complete media to obtain colonies that lost the plasmid bearing a wild-type allele. Such a loss occurs at a much lower rate if the second plasmid contains a mutant rather than another wild-type allele. However, if the wild-type allele is then reintroduced into the same cells and the plasmid loss procedure is repeated, a plasmid with a wild-type allele is lost at the same rate in cells with either another wild-type or mutant allele. This means that a certain amount of stable genetic or epigenetic changes accumulate in the yeast strains during the first round of plasmid shuffling, conferring adaptation to mutations in the release factor genes.

**Figure 2:**
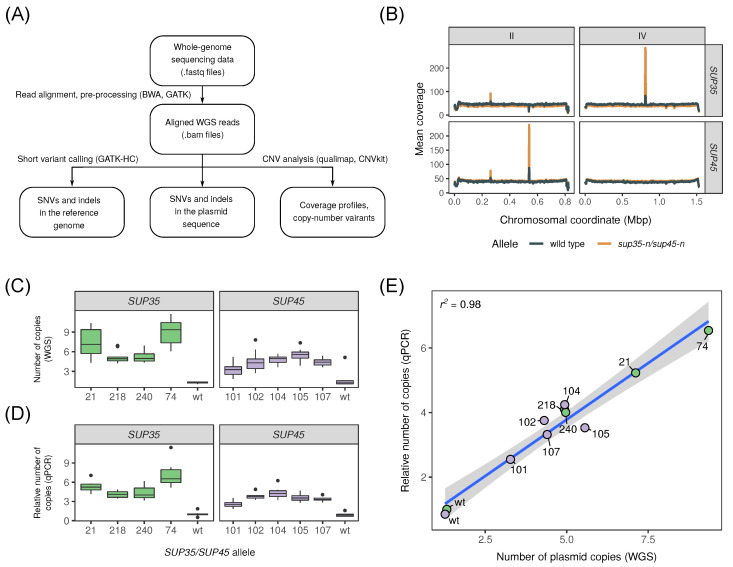
Whole-genome sequencing identifies an increase in the number of copies of centromeric plasmids bearing *sup35-n* or *sup45-n* alleles. (**A**) A schematic representation of the WGS data analysis workflow used in the study. Software tools used to perform each step are indicated in brackets. (**B**) Average profiles of read coverage of chromosomes II and IV for strains bearing wild-type and mutant alleles of *SUP45* or *SUP35*, respectively. Coverage data was collected using qualimap. (**C**,**D**) Numbers of plasmid copies estimated using WGS data (**C**) or qPCR with primers for the *bla* gene (**D**). (**E**) Scatterplot showing the correspondence between estimated plasmid copy number from WGS and qPCR data. Labels correspond to *SUP35* and *SUP45* alleles. Solid line represents the linear regression line; gray envelope corresponds to the confidence interval of the linear regression.

To discover the genetic factors that confer adaptation of yeast cells to mutations in release factors genes, we sequenced a broad set of 100 strains bearing different alleles of both *SUP35* and *SUP45* genes (of these, 4 and 7 strains bore plasmids with the wild-type *SUP35* or *SUP45* alleles, respectively, 34 and 55 contained plasmids with different *sup35-n* and *sup45-n* alleles, respectively). Sequencing reads were aligned onto the reference genome of the corresponding U-1A-D1628 strain [24] or onto the sequence of a plasmid bearing the *SUP35* or *SUP45* copy, and the resulting alignments were used to detect different types of variants in the strains’ genomes (Figure 2A).

We first analyzed the presence of short variants (including single-nucleotide substitutions and short insertions/deletions) in strains bearing wild-type or mutant alleles of release factor genes. To this end, we performed short variant calling using GATK HaplotypeCaller (see Materials and Methods) using reads aligned onto the reference genome or plasmid sequence. We then aggregated the variant calling results and analyzed whether any common variants in both the yeast genome and the plasmid sequence accumulate in strains harboring *sup35-n* and *sup45-n* alleles. Unfortunately, we found no genetic variants in the yeast genome that were observed more than two times in a sample of 89 strains bearing mutant alleles of release factor genes (Supplementary Figure S1), with the exception of the exact mutations that were introduced in each case (i.e., *sup35-n* or *sup45-n*) (Figure S1). When the plasmid sequence was used for variant calling, we found several variants that occurred several times across our set of strains. These included 33 variants in the sequence of plasmids carrying the *sup35-n* alleles, and 41 - in the plasmids carrying the *sup45-n* alleles, which also included *SUP35* and *SUP45* mutations introduced during the experiment (Supplementary Table S1). Most of these variants, however, were false positive heterozygous calls in the sequences shared by plasmid and genomic DNA. Of the remaining variants, several were localized in the bacterial origins and polylinker sequences. However, a small number of backbone-specific plasmid variants were also discovered. Three different substitutions were identified in the backbone of the plasmid carrying the *sup45-105* mutant allele. We found a deletion that occurs in all *sup35-218* samples and localized in the region of the centromere and ARS, but we could not detect it in any plasmids carrying other mutant *sup35-n* alleles. Thus, no functionally significant variants in the plasmid sequence were shared between different mutant strains, indicating these represent variants that accumulated in the sequence of a plasmid during its initial construction rather than in the process of adaptation.

Having found no common single-nucleotide changes in the sequence of the yeast genome or plasmids bearing *sup35-n* and *sup45-n* alleles, we next went on to investigate whether any substantial copy number variants (CNVs) or any other large structural changes occur during the adaptation to the studied mutations. To do so, we first inspected the read coverage profiles across the genome. To our surprise, we found several notable peaks of coverage that occurred systematically in strains bearing different mutant alleles. The coverage peaks were localized at the chromosomes IV and II of the reference genome assembly and occurred in all strains bearing *sup35-n* and *sup45-n* alleles, respectively. No coverage peaks at the corresponding locations were observed in strains bearing wild-type alleles (Figure 2B). Chromosomes II and IV harbor *SUP45* and *SUP35* genes, respectively; this observation suggests that the elevated coverage indicates an increase in the copy number of the corresponding mutant allele.

Increase in the number of gene copies can be explained in two possible ways. First, additional copies of a mutant allele may arise due to multiple recombination events within the same plasmid. Second, the number of copies of the plasmid bearing the corresponding mutant allele may be increased, despite the centromeric nature of the plasmids used in this study. To evaluate both of these hypotheses, we first visually inspected the per-base coverage profiles of the plasmids for strains bearing the *sup35-n* and *sup45-n* mutations. Such an analysis revealed that the sequence coverage is increased across all parts of the plasmid sequence in both *sup35-n* and *sup45-n* mutants (Supplementary Figure S2). These observations indicate that the increase in coverage we observed in the earlier analysis is caused by an increase in the number of copies of the centromeric plasmids bearing mutant *sup35-n* or *sup45-n* alleles. Direct estimation of the number of plasmid copies using WGS data (see Materials and methods) revealed that the estimated number of copies ranged from 4.3 to 11.7 for *sup35-n* and from 1.8 to 7.8 for *sup45-n* mutants (Figure 2C). Notably, the estimated number of copies was higher in strains that lost the wild-type allele spontaneously during cultivation on complete medium (Supplementary Figure S3) compared to strains grown on 5-FOA containing medium used to facilitate plasmid loss.

To validate our WGS-based estimates, we performed qPCR using primers specific for the plasmid-specific beta-lactamase gene (*bla*) using a subset of cells that were selected on 5-FOA continuing media. Our results confirmed the differences in the relative number of the *bla* gene copies between strains carrying wild-type or mutant alleles of *SUP35* and *SUP45* genes (Figure 2D). The number of plasmid copies was increased systematically in strains bearing all studied *sup35-n* and *sup45-n* alleles compared to the respective reference strains. All differences were statistically significant according to the Wilcoxon–Mann–Whitney test (*p* < 0.01). We were pleased to discover that the estimated numbers of plasmid copies obtained using WGS data and qPCR experiments showed a nearly perfect correlation (Pearsons’s *r^2^* = 0.98) (Figure 2E). Taken together, these results suggest that an increase in the plasmid copy number serves as the primary mechanism of adaptation of yeast cells to mutations in release factor genes.

**Figure 3:**
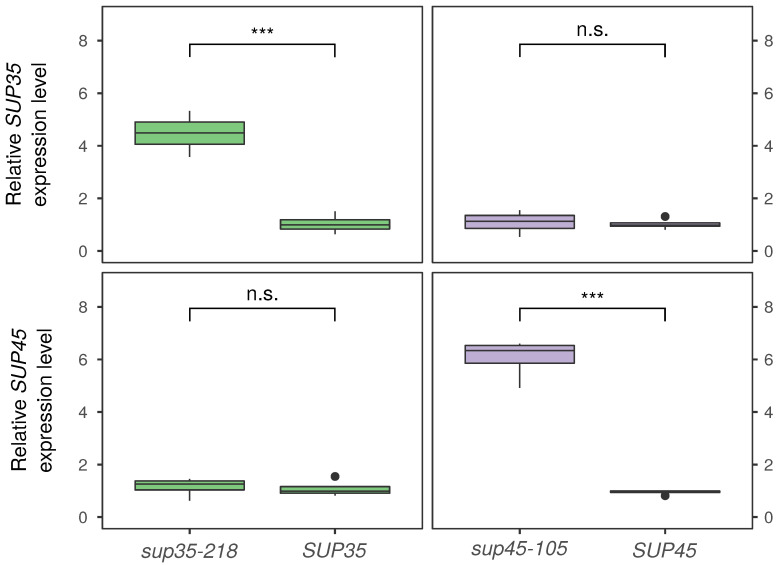
RT-qPCR shows a significant increase in the expression of plasmid-borne mutant *sup35-218* and *sup45-105* alleles. Shown are relative expression levels of the corresponding genes estimated using the ΔΔC_T_ method (see Materials and Methods). *ADH1* was used as the reference gene in all cases. n/s — *p* > 0.05; *** — *p* < 0.001 in the Wilcoxon–Mann–Whitney test.

While the data presented clearly indicate that the increased number of plasmid copies contributes to the adaptation of yeast to *sup35-n* and *sup45-n* mutations, it remained unclear whether an increase in the number of plasmid copies has any effect on the level of expression of mutant alleles respective genes. To answer this question, we performed an expression analysis of *SUP35* and *SUP45* genes using RT-qPCR. Two mutant alleles were selected for this analysis — *sup35-218* and *sup45-105*. In both cases, we observed a steady and significant increase in the level of expression of the plasmid-borne mutant allele (Figure 3). Quite notably, no changes in the expression of wild-type *SUP45* and *SUP35* alleles were observed in cells bearing *sup35-218* and *sup45-105* alleles, respectively, (Figure 3). The results of gene expression analysis indicate that the increase in the number of plasmid copies does indeed translate into increased levels of transcription of mutant alleles, which may in turn aid higher levels of full-length protein production (see Discussion).

Having demonstrated that the number of copies of plasmids bearing *sup35-n* or *sup45-n* alleles increases in all cells bearing such plasmids as a sole source of eRF3 or eRF1, we asked whether similar mechanisms of gene amplification may contribute to the survival of strains bearing *sup35-n* and *sup45-n* alleles as a single chromosomal copy of the corresponding genes. Such strains have also been previously isolated in our group and extensively characterized [11,12].

**Figure 4:**
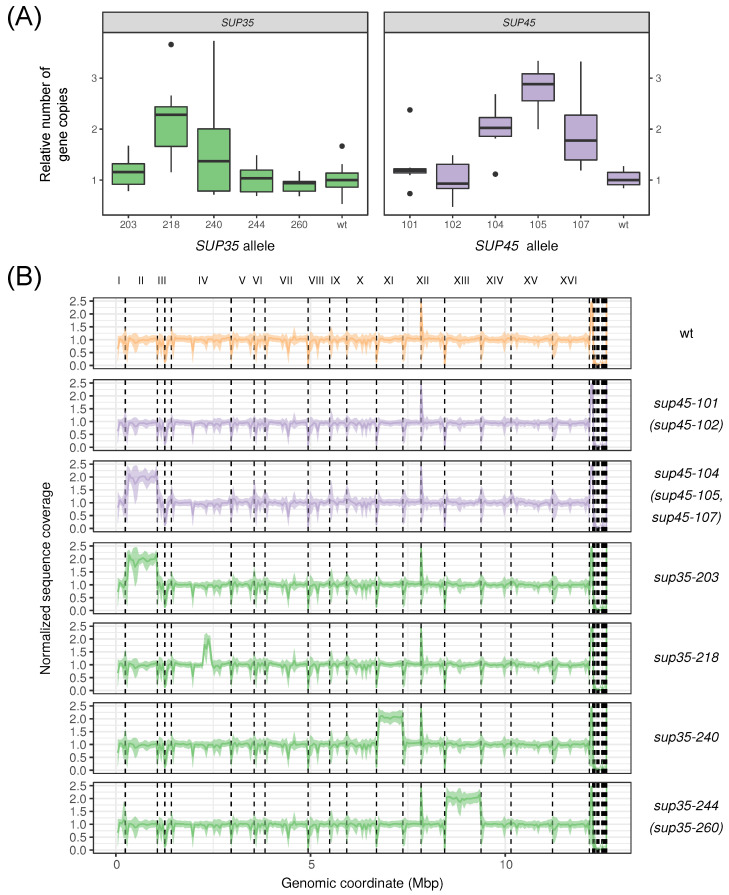
Some of the mutant alleles of *SUP35* and *SUP45* genes are amplified even when located at the normal chromosomal location. (**A**) Boxplot showing the relative number of *SUP35* (left) or *SUP45* (right) gene copies in 1B-D1606 strains according to qPCR results. Number of gene copies was estimated using the ΔΔC_t_ method (see Materials and Methods), (**B**) Normalized sequence coverage profiles across the U-1A-D1628 genome [24]. Dashed lines correspond to contig boundaries. Representative coverage profiles for indicated sets of alleles are shown; strains with similar coverage profiles are indicated in parentheses. Colored envelope corresponds to the standard deviation.

We first used qPCR to compare the number of copies of the *SUP35* and *SUP45* genes in strains carrying wild type or different nonsense mutant variants of these genes on the 1B-D1606 background. qPCR results indicate that the number of mutant gene copies is increased compared to the wild-type control in several of the studied strains (Figure 4A). Specifically, we observed a two-fold increase in the number of copies for the *sup35-218*, *sup45-104*, and *sup45-107* alleles. For *sup45-105*, the observed increase in the copy number was nearly three-fold, though the differences between *sup45-105* and *sup45-104* or *sup45-107* were not statistically significant (*p* > 0.05 in the Wilcoxon–Mann–Whitney test). In the case of the *sup35-240* allele, the results of the qPCR analysis were inconclusive, ranging from one to four gene copies in different replicates. For the remaining mutant alleles tested (*sup35-203*, *sup35-244*, *sup35-260*, *sup45-101*, *sup45-102*) the number of copies did not differ from the wild type (Figure 4A).

Given these findings, we next asked what are the structural genomic changes that lead to the increased number of *SUP35* or *SUP45* gene copies. To answer this question, we performed whole-genome sequencing of 1B-D1606 strain carrying mutant *sup45-n* and *sup35-n* alleles. The obtained WGS data was compared with the reference genome assembly of the PGC strain. In good concordance with the results of qPCR, in some of the strains we observed an increase in coverage of the regions where the *SUP35* and *SUP45* genes are located (Figure 4B, Supplementary Figure S4). However, such an increase was due to different reasons: for example, in case of *sup45-104*, *sup45-105*, and *sup45-107* alleles, we observed a disomy of the entire chromosome II, while for the *sup35-218* mutation, we observed a local duplication of a segment of chromosome IV containing the *SUP35* gene. Interestingly, the boundaries of this duplication were identical to the boundaries of the duplication which was previously seen in case of the *sup35-222* allele ([29] Supplementary Figure S5). Perhaps most surprisingly, we found several cases in which mutant strains were disomic for chromosomes that do not contain release factor genes. For example, in case of the *sup35-240* mutation, for which the results of qPCR were ambiguous, we observed disomy for the chromosome XI. In case of *sup35-203*, we also observed a disomy for chromosome II harboring the *SUP45* gene. Finally, 244-1B-D1606 and 260-1B-D1606 strains bore a disomy for chromosome XIII (Figure 4B). Taken together, these results suggest that adaptation to nonsense mutations in the release factor genes in 1B-D1606 strains involves both amplification of the corresponding allele via local duplication or disomy and duplication of other chromosomes. However, the exact mechanisms of how these changes confer viability of these strains, remain a subject for future investigations.

## 4. Discussion

Recent years have seen a huge amount of published *S. cerevisiae* genome sequences, which have revealed a high level of genetic diversity, phenotypic variation and copy number variation in this species [24,37,38,39,40]. In some of the works, it has been shown that structural changes in the genome (such as CNVs) may have an adaptive role (e.g., [38]), however, it remains unclear if selective pressure may actively facilitate the accumulation of genetic changes. In this work, we show that the introduction of a nonsense mutation in the essential *SUP35* and *SUP45* genes leads to gene amplification in both plasmid and chromosomal DNA (Figure 2 and Figure 4).

Normally, centromeric vectors exist in approximately one copy per cell in yeast [41]. However, it has been shown previously that an increase in the number of centromeric plasmid copies is possible under certain circumstances. This phenomenon was recently used as a basis for creation of the “genetic tug-of-war” (gTOW) system that was used to study dosage-sensitive genes in yeast [42]. Similarly to these studies, we show that a number of plasmid copies may increase in selective conditions and confer adaptation to mutations in release factor genes (Figure 2). In one of the recent works, it has been shown that the *PSH1* gene controls plasmid segregation and copy number [43]. Hence, it may be hypothesized that the activity of this gene contributes to the phenomenon of adaptation investigated in our work.

Recently, a series of publications reported different findings regarding the frequency of aneuploidies in both wild and laboratory yeast isolates. For example, Hose et al. reported widespread presence of copy number variations in wild yeast and suggested that dosage compensation mechanisms buffer the effects of such aneuploidies on the phenotype [44]. However, the buffering hypothesis was subjected to criticism and debated [45,46]. In this work, we demonstrated that chromosome copy number changes are not only present in the genomes of laboratory yeast strains [28], but may rapidly accumulate during selection (in this case, upon introduction of nonsense mutations into the release factor genes) (Figure 4). Previously, several similar observations of aneuploidies associated with nonsense suppression and release factor genes have been reported. These include a phenomenon of reversible nonsense suppressor phenotype switching due to chromosome II copy number observed in the presence of missense mutations in the *SUP35* and *SUP45* genes [40]. This case is reminiscent of the chromosome II disomy, which we observed in this work in the *sup35-203* mutant. Another example is the chromosome VIII disomy compensating for the suppressor phenotype in strain with native *SUP35* substituted for its ortholog from *Pichia methanolica* [47]. Interestingly, the amplification of the same chromosome may arise as an adaptation to high copper levels, as it harbors *CUP1* genes [48]. An increase in the chromosome II copy number can also be adaptive, as it was shown to ameliorate the toxicity of polyglutamine, presumably, by increasing *SUP45* expression [49]. Similar reasons may underlie the advantages of sustaining additional chromosome copies in the strains analysed in this work, however, a comparison between aneuploids and their respective euploid counterparts should be made to address this point.

Besides the general role of CNVs and aneuploidies in adaptation, it is also important to consider the possible mechanisms that explain the beneficial effects of gene amplification upon introduction of nonsense mutations in release factor genes. In this study, we show that gene amplification in strains carrying mutant alleles *sup35-n* and *sup45-n* is systematic and likely represents the way of adaptation to translation termination defects. Despite the high number of gene copies present in most mutants, cells bearing mutant alleles still have a decreased levels of vital eRF1 or eRF3 [11]; hence, a certain set of additional factors should contribute to their viability. It is known that full-length eRF1 and eRF3 proteins are synthesized due to nonsense suppression in all mutants bearing *sup35-n* or *sup45-n* alleles. Nonsense suppression efficiency may be influenced by tRNA level, type of stop codon, and the nucleotide context surrounding the termination codon, especially the nucleotide which follows the stop codon [11,18]. All cells bearing *sup45-n* mutations are characterized by an increased content of tRNA fractions potentially capable of recognizing stop codons (i.e., ${\mathrm{tRNA}}_{\mathrm{GUA}}^{\mathrm{Tyr}}$, ${\mathrm{tRNA}}_{\mathrm{CCA}}^{\mathrm{Trp}}$, ${\mathrm{tRNA}}_{\mathrm{UUG}}^{\mathrm{Gln}}$) [50,51]. It is assumed that these tRNAs are involved in the synthesis of the full-length eRF1 protein in strains carrying *sup45-n* mutations. Notably, the total level of all tRNAs differ among mutants and was 1.5- to 2-fold higher in *sup45-105* mutant than in *sup45-101* mutant [20]. This fact allowed us to propose that each *sup45-n* mutant has its own “optimized level of suppression”. The highest level of readthrough was shown for *sup45-102* mutation (20–30%), and the lowest for *sup45-105* (5–8%) [52]. These data might explain our observation that the gene amplification phenomenon is least pronounced for these alleles (Figure 2 and Figure 4). In the case of *sup35-n* mutants, prior studies also revealed an increase in the level of tRNAs [20]. All of the *sup35-n* mutants contain extremely low levels of full-length eRF3. For example, *sup35-21* and *sup35-218* mutants contained only 3% and 8% of full-length eRF3, respectively, compared to the wild-type cells. Notably, *sup35-244* contained the lowest amount of eRF3, equivalent to only 0.5% of the level found in wild-type cells [12]. Despite such a low level of full-length protein, the *sup35-244* allele is not amplified in the genome of the 244-1B-D1606 strain. It is possible that changes in the expression of other genes located on the duplicated chromosome XIII in this strain play a role in the adaptation to low eRF3 levels. However, additional experiments are needed to characterize gene expression in strains with duplicated chromosomes.

All of the aforementioned data support the previously proposed model which supposes the existence of feedback-regulated readthrough of the premature termination codon [19,53]. According to this model reductions in full-length protein promote tRNA-mediated stop codon readthrough, which, in turn, drives partial production of full-length eRF1. In such a model, an increased level of suppressor tRNA or decreased expression of the *SUP45* gene leads to increased readthrough levels. At the same time, negative feedback decreases the readthrough levels upon increased production of the *sup45-n* mRNA, but does not eliminate additional production of full-length eRF1 protein. Hence, we may hypothesize that the increase in the number of gene copies observed in our strains allows for increased levels of *sup35-n* (*sup45-n*) mRNA that provides the optimized level of full-length protein production to support viability.

Different hypotheses might be made regarding the mechanisms of gene amplification upon introduction of *sup35-n* or *sup45-n* alleles. For example, the introduction of mutant *sup35* and *sup45* alleles might lead to genome instability (as already shown in [54]), which in turn increases the number of plasmid and/or chromosome copies. An alternative hypothesis implies that a yeast cell experiences pronounced stress when the translation termination is impaired, leading to incorrect segregation of plasmids and/or chromosomes. During this period of enhanced genomic instability, some cells acquire additional copies of plasmid DNA or an additional chromosomal copy of a gene via aneuploidy or intrachromosomal rearrangement. As a consequence, such cells gain selective advantage and spread in the population. If true, this mechanism provides an additional example of an adaptive mutagenesis phenomenon which might occur under specific selective conditions (reviewed in [29]). However, the correctness of the aforementioned model of gene amplification in *sup35-n* and *sup45-n* mutants remains unproven and predicates the need for further investigations.

## Figures and Tables

**Table 1:** Yeast strains used in this work.

Strajn	Genotype	References	Sequencing Data
U-1A-D1628	*MATα ade1-14(UGA) trp1-289(UAG)*	[11]	[24]
	*his3-Δ200 lys2 ura3-52 leu2-3,112*		
	*SUP45::HIS3MX* [pRS316-SUP45]		
L-1A-D1628	*MATα ade1-14(UGA) trp1-289(UAG)*	[11]	This work
	*his3-Δ200 lys2 ura3-52 leu2-3,112*		
	*SUP45::HIS3MX* [pRS315-SUP45]		
nL-1A-D1628	*MATα ade1-14(UGA) trp1-289(UAG)*	[11]	This work
	*his3-Δ200 lys2 ura3-52 leu2-3,112*		
	*SUP45::HIS3MX* [pRS315-sup45-n]		
U-14-D1690	*MATα ade1-14(UGA) trp1-289(UAG)*	This work	This work
	*his3-Δ200 lys2 ura3-52 leu2-3,112*		
	*SUP35::HIS3MX* [pRSU2]		
L-14-D1690	*MATα ade1-14(UGA) trp1-289(UAG)*	This work	This work
	*his3-Δ200 lys2 ura3-52 leu2-3,112*		
	*SUP35::HIS3MX* [pRSU1]		
nL-14-D1690	*MATα ade1-14(UGA) trp1-289(UAG)*	This work	This work
	*his3-Δ200 lys2 ura3-52 leu2-3,112*		
	*SUP35::HIS3MX* [pRSU1-n]		
1B-D1606	*MATα ade1-14(UGA) his7-1(UAA)*	[11]	[28]; This work
	*lys9-A21(UAA) trp1-289(UAG) ura3-52*		
	*leu2–3,112 gal10-1B*		
101-1B-D1606	*MATα ade1-14(UGA) his7-1(UAA)*	[11]	This work
	*lys9-A21(UAA) trp1-289(UAG) ura3-52*		
	*leu2–3,112 gal10-1B sup45-101*		
102-1B-D1606	*MATα ade1-14(UGA) his7-1(UAA)*	[11]	This work
	*lys9-A21(UAA) trp1-289(UAG) ura3-52*		
	*leu2–3,112 gal10-1B sup45-102*		
104-1B-D1606	*MATα ade1-14(UGA) his7-1(UAA)*	[11]	This work
	*lys9-A21(UAA) trp1-289(UAG) ura3-52*		
	*leu2–3,112 gal10-1B sup45-104*		
105-1B-D1606	*MATα ade1-14(UGA) his7-1(UAA)*	[11]	This work
	*lys9-A21(UAA) trp1-289(UAG) ura3-52*		
	*leu2–3,112 gal10-1B sup45-105*		
107-1B-D1606	*MATα ade1-14(UGA) his7-1(UAA)*	[11]	This work
	*lys9-A21(UAA) trp1-289(UAG) ura3-52*		
	*leu2–3,112 gal10-1B sup45-107*		
222-1B-D1606	*MATα ade1-14(UGA) his7-1(UAA)*	[12]	[29]
	*lys9-A21(UAA) trp1-289(UAG) ura3-52*		
	*leu2–3,112 gal10-1B sup35-222*		
203-1B-D1606	*MATα ade1-14(UGA) his7-1(UAA)*	[12]	This work
	*lys9-A21(UAA) trp1-289(UAG) ura3-52*		
	*leu2–3,112 gal10-1B sup35-203*		
218-1B-D1606	*MATα ade1-14(UGA) his7-1(UAA)*	[12]	This work
	*lys9-A21(UAA) trp1-289(UAG) ura3-52*		
	*leu2–3,112 gal10-1B sup35-218*		
240-1B-D1606	*MATα ade1-14(UGA) his7-1(UAA)*	[12]	This work
	*lys9-A21(UAA) trp1-289(UAG) ura3-52*		
	*leu2–3,112 gal10-1B sup35-240*		
244-1B-D1606	*MATα ade1-14(UGA) his7-1(UAA)*	[12]	This work
	*lys9-A21(UAA) trp1-289(UAG) ura3-52*		
	*leu2–3,112 gal10-1B sup35-244*		
260-1B-D1606	*MATα ade1-14(UGA) his7-1(UAA)*	[12]	This work
	*lys9-A21(UAA) trp1-289(UAG) ura3-52*		
	*leu2–3,112 gal10-1B sup35-260*		

**Table 2:** Plasmids used in this work.

Plasmid	Description (Selectable Marker, Promoter, Gene)	References
pRS316-SUP45	*URA3, P_SUP45_, SUP45*	[30]
pRS315-SUP45	*LEU2, P_SUP45_, SUP45*	[30]
pRS315-sup45-101	*LEU2, P_SUP45_, sup45-101* (G796T) (E266[UAA])	[11]
pRS315-sup45-102	*LEU2, P_SUP45_, sup45-102* (T159A) (Y53[UAA])	[11]
pRS315-sup45-104	*LEU2, P_SUP45_, sup45-104* (T848A) (L283[UAA])	[11]
pRS315-sup45-105	*LEU2, P_SUP45_, sup45-105* (G1153T) (E385[UAA])	[11]
pRS315-sup45-107	*LEU2, P_SUP45_, sup45-107* (T950G) (L317[UGA])	[11]
pRSU2	*URA3, P_SUP35_, SUP35*	[25]
pRSU1	*LEU2, P_SUP35_, SUP35*	[25]
pRSU1-21	*LEU2, P_SUP35_, sup35-21* (C1264T) (Q422[UAA])	[12]
pRSU1-74	*LEU2, P_SUP35_, sup35-74* (C388E) (Q130[UAA])	[12]
pRSU1-218	*LEU2, P_SUP35_, sup35-218* (G541T) (E181[UAA])	[12]
pRSU1-240	*LEU2, P_SUP35_, sup35-240* (C166T) (Q56[UAA])	[12]

Full annotated maps of pRS315-SUP45 and pRSU1 plasmids are available in Supplementary File S1.

**Table 3:** Primers used in this work.

No.	Primer Name	Target Gene	Sequences
1	bla_F	*bla*	ATAAATCTGGAGCCGGTGAG
2	bla_R	*bla*	CTACGATACGGGAGGGCTTA
3	SUP45_F	*SUP45*	CGATCCAAGACTAGCATGTAAG
4	SUP45_R	*SUP45*	CTTGAACATACTTGACATTGGC
5	SUP35_F	*SUP35*	ACAACAAGGTAACAACAGATACC
6	SUP35_R	*SUP35*	GGATTGAATTGCTGCTGATAAC
7	ACT1_F	*ACT1*	TAACGGTTCTGGTATGTGTAAAGC
8	ACT1_R	*ACT1*	GCTTCATCACCAACGTAGGAGTC
9	F-ADH1-RT	*ADH1*	CAAGTCGTCAAGTCCATCTC
10	R-ADH1-RT	*ADH1*	GTAGACAAGCCGACAACCT

## Data Availability

All code pertinent to the bioinformatic or statistical analysis presented in this work is available at https://github.com/mrbarbitoff/yeast_cnv_analysis/. All sequencing data have been submitted to NCBI Sequencing Read Archive and will be available shortly.

## Supplementary Materials

The following are available online at https://www.mdpi.com/article/10.3390/genes12122019/s1, Figure S1: A scatter plot representing the results of variant calling against the reference genome sequence of the U-1A-D1628 strain, Figure S2: Coverage distribution in mutant and wild-type strains across plasmid sequences, Figure S3: Plasmid copy number estimation in strains that were selected using 5-FOA or spontaneously lost the plasmid containing the wild-type *SUP45* allele, Figure S4: Normalized sequence coverage profiles for all 1B-D1606 strain derivatives analysed, Figure S5: Comparison of the chromosome IV coverage profiles for the 218-1B-D1606 and 222-1B-D1606 sequences aligned onto the 74-D694 genome, Table S1: Variants in the plasmids carrying mutant alleles *sup35-n* and *sup45-n*, File S1: A ZIP archive containing plasmid sequences in the SnapGene format.

## Sample Availability

Yeast strains and plasmids used in the work are available from the authors.

## Abbreviations

The following abbreviations are used in this manuscript:

PGC	Peterhof Genetic Collection
WGS	Whole genome sequencing
eRF1/eRF3	eukaryotic release factor 1/3
5-FOA	5-fluoroorotic acid
