# Supplementary material for: Gene Amplification as a Mechanism of Yeast Adaptation to Nonsense Mutations in Release Factor Genes
Source: Genes (Basel). 2021 Dec 19;12(12):2019. doi: 10.3390/genes12122019 (PMC8701342; doi:10.3390/genes12122019)
Supplement: Supplementary file 1 [file genes-12-02019-s001.zip › 2021-dec-12 - Yeast CNV paper SI.pdf]

Gene amplification as a mechanism of yeast adaptation to nonsense mutations in release factor genes

Evgeniia M. Maksiutenko[1,2], Yury A. Barbitoff[1,3], Andrew G. Matveenko[1], Svetlana E. Moskalenko[1,2], Galina A. Zhouravleva[1,4]

[1] - Dpt. of Genetics and Biotechnology, St. Petersburg State University, St. Petersburg, Russia
[2]- St. Petersburg Branch, Vavilov Institute of General Genetics of the Russian Academy of Sciences, St. Petersburg, Russia
[3] - Bioinformatics Institute, St. Petersburg, Russia
[4] - Laboratory of Amyloid Biology, St. Petersburg State University, St. Petersburg, Russia

Supplementary Tables

Table S1. Variants in the plasmids carrying mutant alleles *sup35-n* and *sup45-n*. Position and region annotations conform to the pRSU1 and pRS315-SUP45 maps (Supplementary File S1) in cases of *sup35* and *sup45* alleles, respectively.

Allele	Type	Substitution	Position	Region	Number of clones
sup35-n	Insertion	T > TCTAG	5110	MCS	34
sup35-21	SNP	C > T	2964	*SUP35* ORF	9
sup35-74	SNP	C > T	2088	*SUP35* ORF	8
sup35-74	SNP	A > G	2391	*SUP35* ORF	8
sup35-74	SNP	A > G	2594	*SUP35* ORF	8
sup35-74	SNP	T > C	2800	*SUP35* ORF	8
sup35-74	SNP	T > C	10205	Bacterial ori	8
sup35-218	SNP	G > T	2241	*SUP35* ORF	9
sup35-218	Deletion	TTA > T	3849	Unannotated region	2
sup35-218	Deletion	GA > G	8463	ARSH4	9
sup35-218	SNP	A > C	10117	Bacterial ori	7

sup35-240	SNP	C > T	1866	*SUP35* ORF	8
sup35-240	Deletion	TTA > T	3849	Unannotated region	1
sup45-n	SNP	A > G	1715	*SUP45* ORF	55
sup45-n	SNP	G > T	2165	*SUP45* ORF	55
sup45-n	SNP	T > C	2333	*SUP45* ORF	55
sup45-n	SNP	T > A	2348	*SUP45* ORF	55
sup45-n	SNP	G > A	2573	*SUP45* ORF	55
sup45-n	SNP	T > C	2654	*SUP45* ORF	55
sup45-101	SNP	A > T	1651	*SUP45* ORF	2
sup45-101	SNP	G > T	2442	*SUP45* ORF	11
sup45-102	SNP	T > A	1805	*SUP45* ORF	11
sup45-104	SNP	T > A	2494	*SUP45* ORF	11
sup45-105	SNP	A > T	891	*SUP45* promoter	1
sup45-105	SNP	G > T	2799	*SUP45* ORF	11
sup45-105	SNP	G > A	3380	Unannotated region	1
sup45-105	SNP	G > A	6151	Unannotated region	1
sup45-105	Deletion	AT > A	6352	*LEU2* terminator	1
sup45-107	SNP	T > G	2596	*SUP45* ORF	11

Supplementary Figures

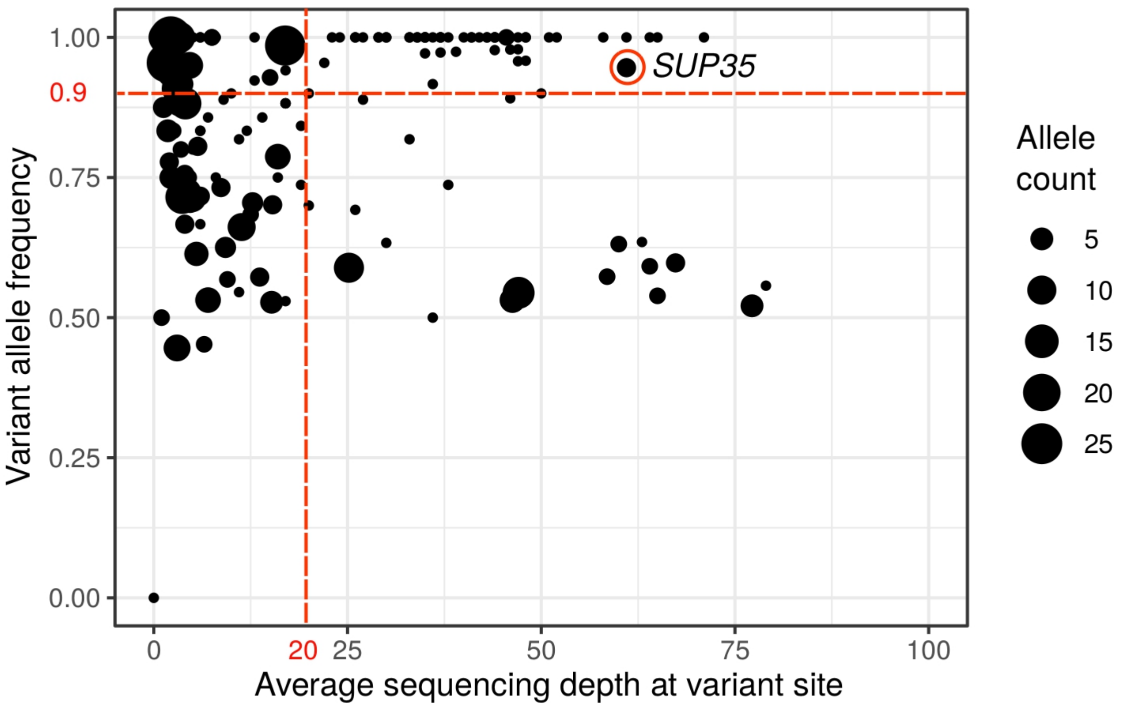

Figure S1. A scatter plot representing the results of variant calling against the reference genome sequence of the U-1A-D1628 strain. Each dot represent a variant site, size of the dot is proportional to the variant allele count in the set of 8

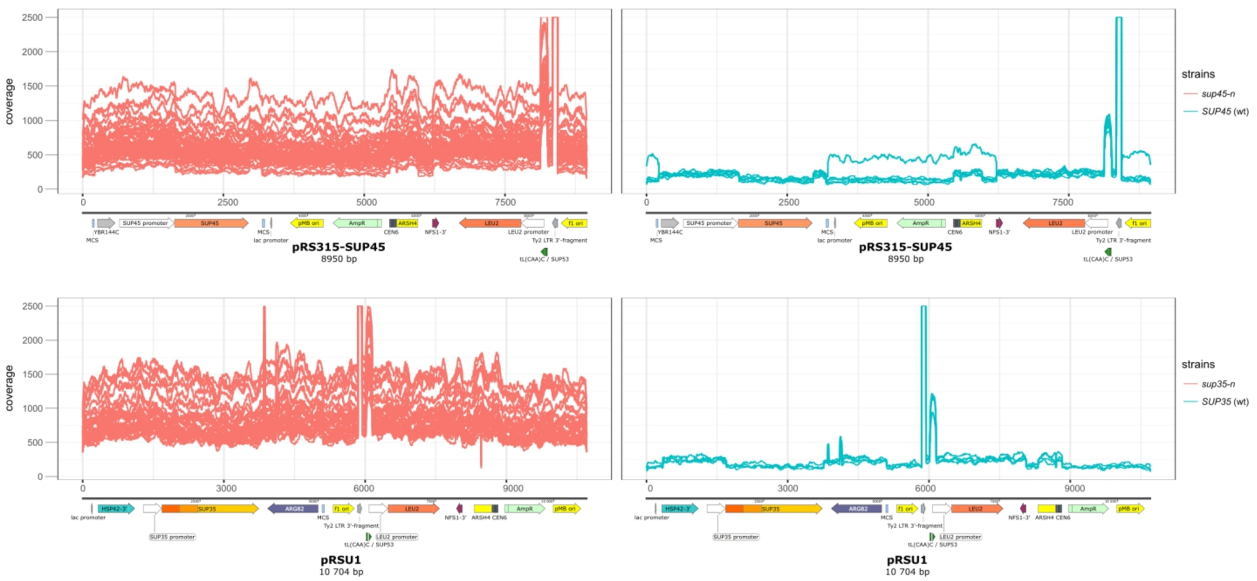

Figure S2. Coverage distribution in mutant and wild-type strains across plasmid sequences.

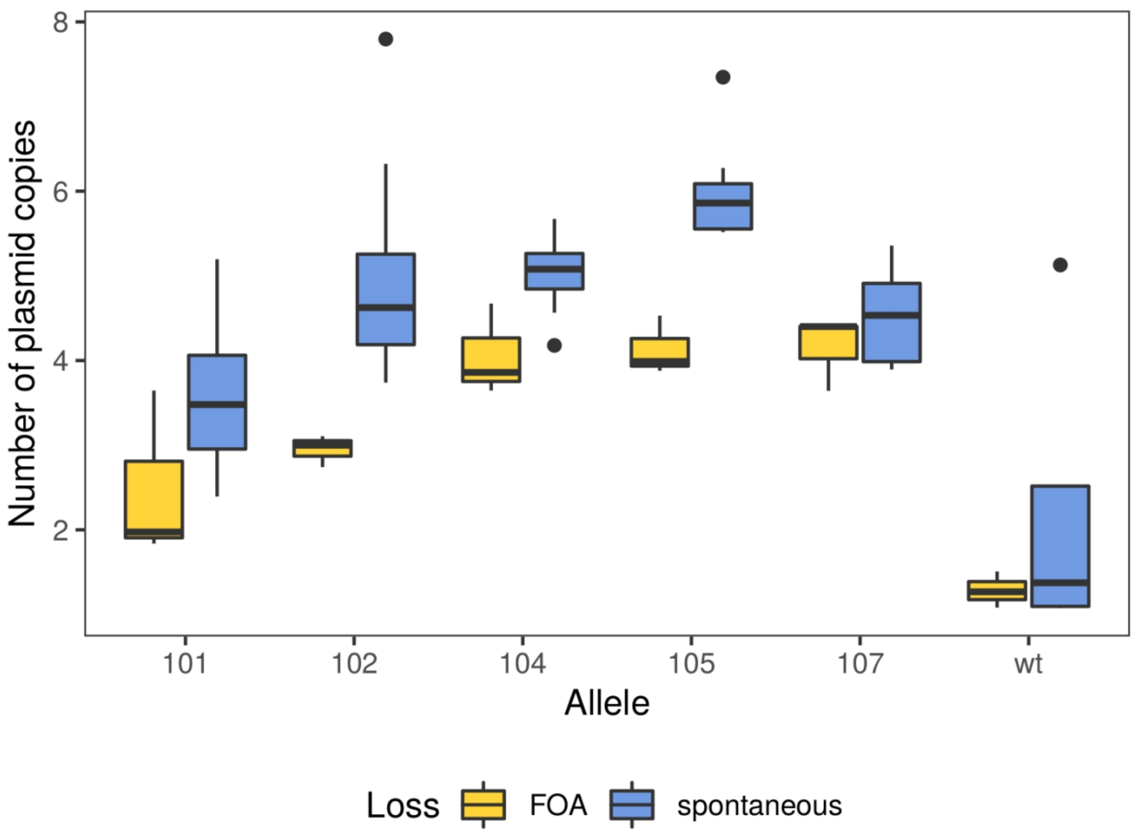

Figure S3. Plasmid copy number estimation in strains that were selected using 5-FOA (yellow) or spontaneously lost the plasmid containing the wild-type *SUP45* allele (blue).

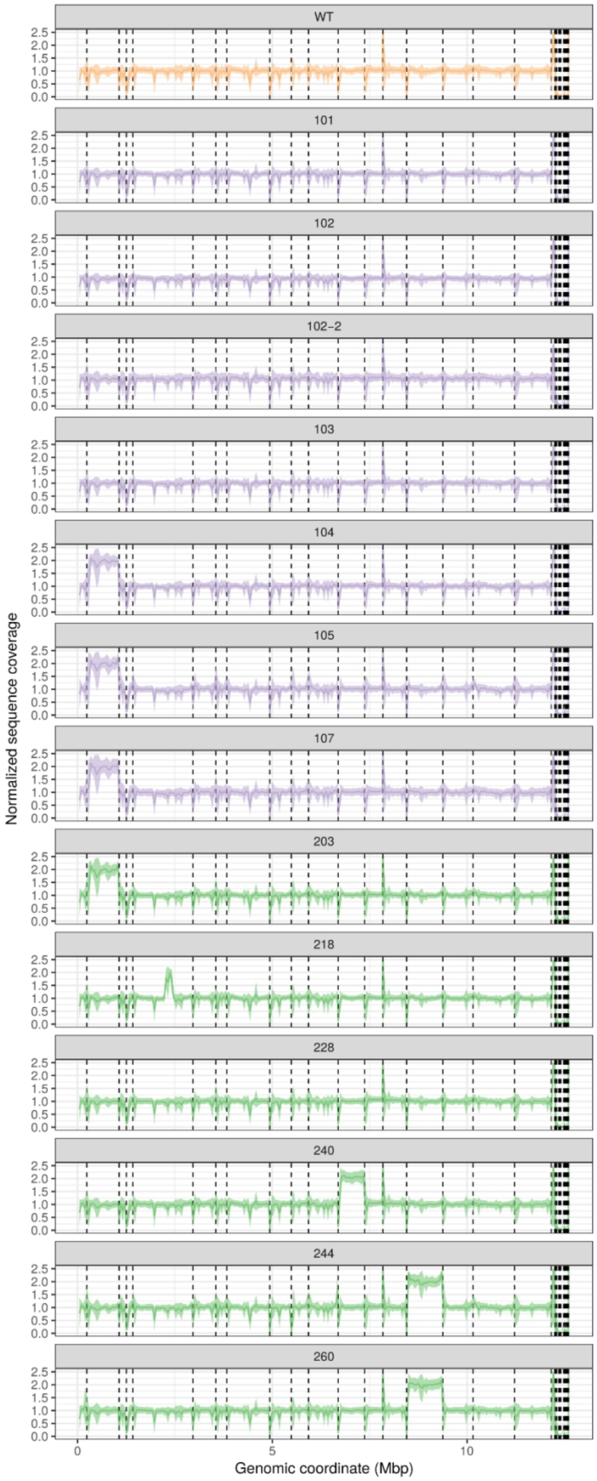

Figure S4. Normalized sequence coverage profiles for all 1B-D1606 strain derivatives analysed. Color corresponds to the mutant gene (orange - wild-type, purple - *sup45-n*, green - *sup35-n*) Dashed lines correspond to contig boundaries. Two clones from two different sources with confirmed *sup45-102* allele (denoted as "102" and "102-2") were analyzed.

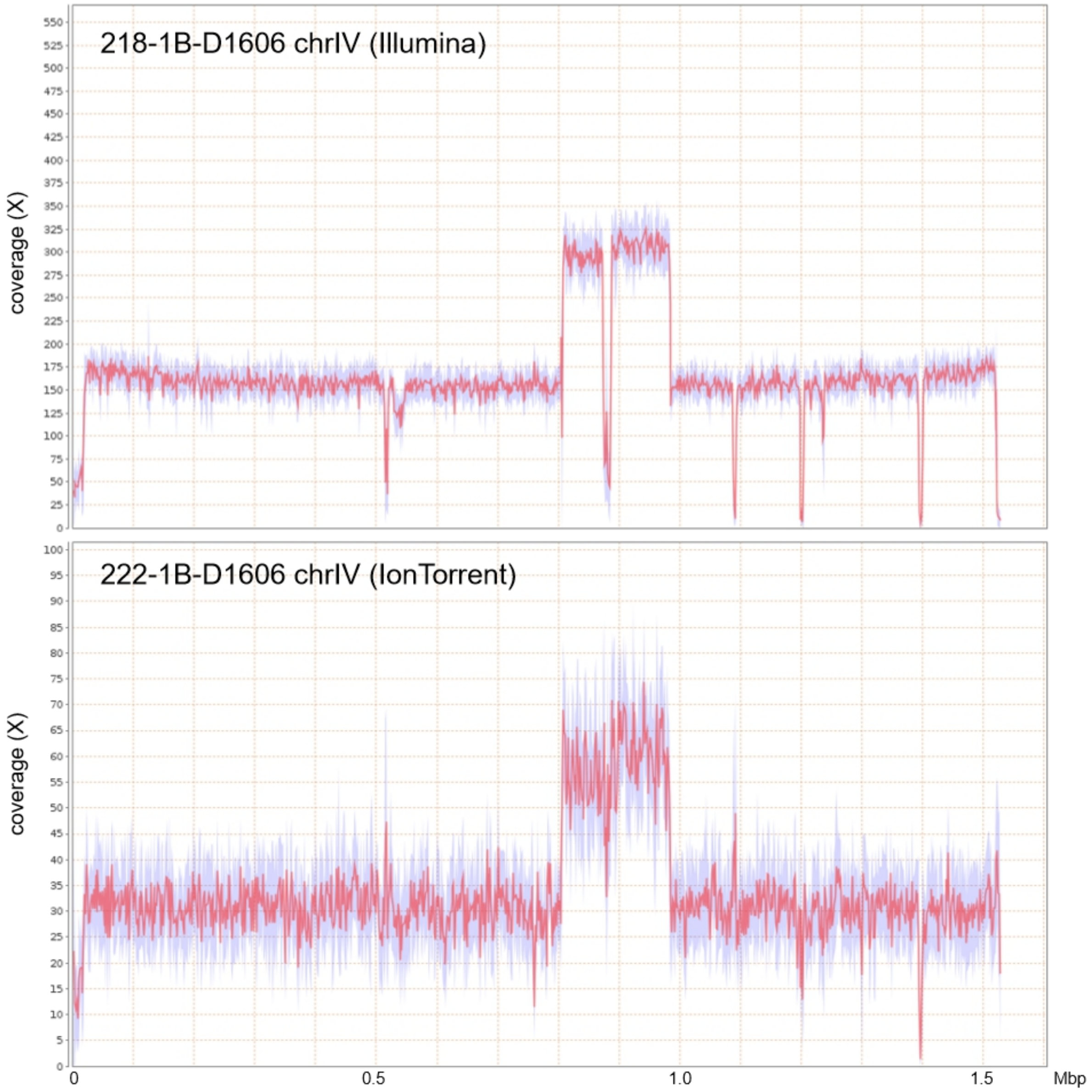

Figure S5. Comparison of the chromosome IV coverage profiles for the 218-1B-D1606 and 222-1B-D1606 sequences aligned onto the 74-D694 genome (Barbitoff et al., 2021).

Supplementary File S1. A ZIP archive containing plasmid sequences in the SnapGene format.
